# UNet with Attention Networks: A Novel Deep Learning Approach for DNA Methylation Prediction in HeLa Cells

**DOI:** 10.3390/genes16060655

**Published:** 2025-05-28

**Authors:** Apoorva, Vikas Handa, Shalini Batra, Vinay Arora

**Affiliations:** 1 Department of Biotechnology, Thapar Institute of Engineering & Technology, Patiala 147004, India; vikas.handa@thapar.edu; 2 Department of Computer Science & Engineering, Thapar Institute of Engineering & Technology, Patiala 147004, India; sbatra@thapar.edu (S.B.); vinay.arora@thapar.edu (V.A.)

**Keywords:** DNA methylation, uterine cervical cancer, epigenetics, deep learning, neural networks, machine learning

## Abstract

**Background**: The purpose of the proposed study is to investigate the efficacy of UNet in predicting Deoxyribonucleic Acid methylation patterns in a cervical cancer cell line. The application of deep learning to analyse the factors affecting methylation in the context of cervical cancer has not yet been fully explored. **Methods**: A comprehensive performance evaluation has been conducted based on multiple window sizes of DNA sequences. For this purpose, three different parameter-analysis techniques, namely, autoencoders, Generative Adversarial Networks, and Multi-Head Attention Networks, were used. This work presents a novel framework for methylation prediction in promoter regions of various genes. **Results and Conclusions**: Experimental results have proved that attention networks in association with UNet achieved a significant accuracy level of 91.01% along with a sensitivity of 89.65%, specificity of around 92.35%, and an area under curve of 0.910 on ENCODE database. The proposed model outperformed three state-of-the-art models: Convolutional Neural Network, Transfer Learning, and Feed Forward Neural Network with K-Nearest Neighbour. Moreover, validation of the model in five gene promoters achieved an accuracy of 81.60% with an area under curve score of 0.814, a *p*-value of $3.62\times 10^{-19}$, and Cohen’s Kappa value of 0.631. This novel approach has led to a better understanding of epigenetic variables and their implications in cervical cancer, offering potential insights into therapeutic strategies.

## 1. Introduction

The intricate interplay between genetics and epigenetics governs cellular functions, while Post-Translational Modifications (PTMs) have a pivotal role in regulating gene expression. Epigenetic modifications, including Deoxyribonucleic Acid (DNA) methylation, have emerged as crucial regulators of cellular processes, influencing normal development and disease progression [1]. This research embarks on a journey into the realm of epigenetics, focusing on the interplay between DNA methylation, particularly Cytosine-phosphorylated Guanine (CpG) methylation, and its intricate relationship with cancer, with a specific emphasis on cervical cancer.

**Figure 1:**
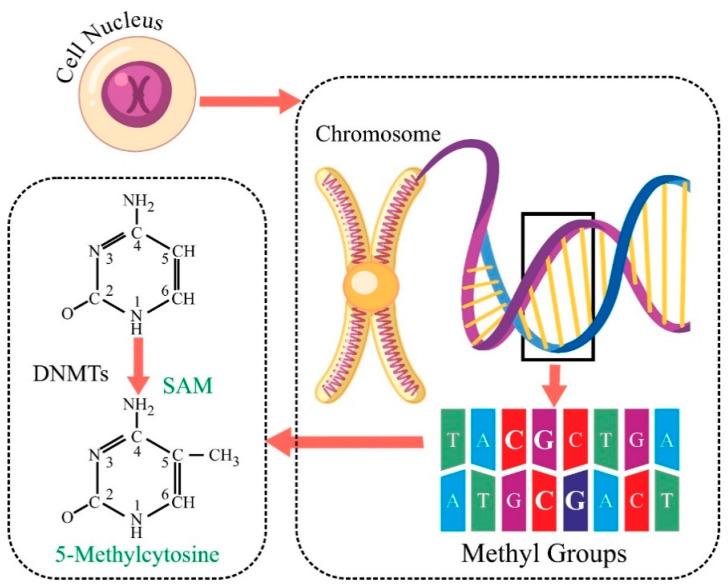
Mechanism of addition of methyl group at Cytosine base in a DNA strand.

Among the various epigenetic modifications, DNA methylation, specifically CpG methylation, has garnered substantial attention in the context of cancer biology [2]. DNA methylation, which involves adding methyl groups to Cytosine residues in CpG dinucleotides, is a well-recognised epigenetic modification implicated in cancer pathogenesis, as shown in Figure 1 [3]. Aberrant DNA methylation patterns can lead to the dysregulation of gene expression, promoting tumorigenesis [4]. Existing literature has highlighted the correlation between altered DNA methylation and certain cancer types, including cervical cancer [5]. The intricate interplay between CpG methylation and cancer opens a window of opportunity for understanding the molecular underpinnings of cervical carcinogenesis, paving the way for improved diagnostic strategies.

Cervical cancer is a prominent example wherein epigenetic alterations are crucial to the onset and course of the disease. Persistent infection with Human Papilloma Virus (HPV) is one of the well-known precursors for cervical cancer, and epigenetic modifications further contribute to the transformation of normal cervical cells into malignant counterparts [6]. Despite recent advancements in diagnostics and prevention, cervical cancer remains a leading cause of mortality among women worldwide. This research delves into the epigenetic landscape of cervical cancer, focusing on unravelling the specific DNA methylation patterns that serve as prospective biomarkers for prediction and early detection.

The current landscape of cancer diagnosis relies heavily on comprehensive molecular profiling, integrating data from various high-throughput techniques. Databases such as the Cancer Genome Atlas (TCGA) and the Encyclopaedia of DNA Elements (ENCODE), along with sequencing experiments, play a crucial role in deciphering genomic and epigenomic alterations associated with cancer [7]. Conventional machine learning (ML) methods require the creation of specific feature designs, a process that demands expertise and lengthy experimentation along with substantial financial resources to identify effective feature descriptors. These characteristics collectively impede their widespread application.

Moreover, despite the advancements, existing diagnostic approaches often lack the precision required for early detection and personalised treatment strategies. The sheer volume and complexity of data necessitate more sophisticated tools for accurate interpretation. Bioinformatics tools and simulation models have been instrumental in interpreting large-scale molecular data in cancer research. Pattern-based predictions employing ML algorithms offer a promising avenue for overcoming the limitations of traditional bioinformatics tools [8]. The identification of specific DNA methylation patterns as biomarkers holds the potential to enhance diagnostic accuracy (*ACC*) and prognostic precision, providing a more comprehensive understanding of the epigenetic changes associated with cervical cancer. Computational algorithms, including decision trees, Support Vector Machines (SVMs), and neural networks, have been proposed to handle the intricate data complexity inherent in epigenetic studies [9]. These algorithms enable the identification of subtle patterns that may elude traditional analyses, thereby improving the robustness of diagnostic predictions.

### 1.1. Scope of the Study

This research aims to explore DNA methylation, an important epigenetic modification, with a specific focus on DNA methylation patterns in cervical cancer. The aim is to unravel methylation patterns for early diagnosis and prognosis by leveraging state-of-the-art computational algorithms and integrating diverse datasets. This work contributes to the refinement of current diagnostic approaches, fostering a deeper understanding of epigenetic mechanisms underlying cervical cancer and paving the way for personalised therapeutic intervention.

### 1.2. Contribution of the Study

The proposed work contributes significantly in the following manner:

**Influence of Feature Selection Methods:** The research outlines the frameworks of autoencoders, generative neural networks, and attention networks to analyse the role of features associated with DNA methylation. By combining the capabilities of attention networks with the UNet architecture, this research introduces a novel approach that enhances predictive *ACC* of DNA methylation patterns.

**Significance of Window Size**: The research conducts a comprehensive performance evaluation using multiple window sizes of DNA sequences. This analysis highlights the significance of the association of sequence length of 500 bp with DNA motifs for the prediction of DNA methylation.

**Influence of Methylation Patterns in Cancerous and Non-cancerous Cell Lines:** The proposed work showcases the difference between methylation patterns of human embryonic stem cells, leukaemia, and liver cancer cells as indicated by evaluation of architecture developed on the basis of cervical cancer samples for DNA methylation prediction.

**Significance of Methylation Prediction and Statistical Analysis in Promoter Regions:** The proposed work presents a novel approach to understanding the importance of DNA methylation in the promoter region of various genes associated with cervical carcinoma. This has been statistically analysed with the help of the chi-square test and measurement of Cohen’s Kappa (κ) to establish the robustness of this study.

## 2. Related Works

### 2.1. Machine Learning Methods

Accurate prognosis prediction for patients with cancer is of utmost significance for personalised treatment and mortality reduction. Several studies have shown that ML and Deep Learning (DL) algorithms have made great contributions to the successful diagnosis of malignancies. Table 1 enlists prior reports demonstrating the effectiveness of different algorithms used to predict DNA methylation. In 2006, Fang et al. utilised SVMs for the prediction of CpG island methylation in human brains. They achieved an *ACC* of approximately 84.52%. However, the experiment was hampered by the scarcity of data as the majority of tissues considered for experimental purposes had less than ten samples [10]. Previti et al. employed decision-tree classifiers to forecast the methylation of CpG sites specific to tissues in a normal human embryonic stem cell line. The Area Under the Receiver Operating Characteristic (AUROC) curve indicated that their forecasts had an accuracy of 82.00% [11].

Similarly, Jiang et al. introduced a model called Light CpG that utilised a gradient-boosting gradient tree and RF classifier to identify CpG methylation in individual cells. This model achieved an impressive AUROC of 0.921 in the HepG2 cell line [12]. In 2021, Luca Bedon et al. developed a model using epigenetic ML methods such as random forest to monitor hepatocellular carcinoma progression by analysing genome-wide DNA methylation profiles [13]. Based on 34 epigenetic probes, it achieved a significant *ACC* of 80% and predictive power, effectively stratifying patients into both low- and high-risk categories for progression, confirmed by survival and decision curve analyses. ML techniques have shown greater effectiveness and reduced expenses in comparison to wet laboratory experiments. However, the manual selection of feature descriptors and classifiers, based on the researchers’ experience, limited the efficacy of ML models.

### 2.2. Deep Learning Methods

In order to improve the *ACC* of prognosis predictions represented by a Brier score of 0.21 in DNA methylation data, Tian et al. followed an autoencoder-based DL approach to determine the two prognostic subgroups of gliomas utilising Ribonucleic Acid (RNA) sequencing and DNA methylation data [14]. The model showcased greater performance in both TCGA and Chinese Glioma Genome Atlas (CGGA) datasets, with a high C-index and significant log-rank *p*-values, and identified 389 DNA methylation-driven genes significantly enriched in key biological pathways. In 2022, Amor et al. introduced an innovative deep-embedded refined clustering approach based upon autoencoders to differentiate breast cancer types using DNA methylation, aiming to optimise dimensionality reduction and identification in tandem [15]. With an error rate of 0.73% and an independent clustering *ACC* of 0.9927 on 137 tissues from breast cancer, this study showed significant ACC. Though it outperformed existing state-of-the-art techniques under similar specifications, it reduced dimensionality up to 99.96% with the use of only ten features, which led to the loss of some biologically important informative features.

Angermueller et al. employed a Convolutional Neural Network (CNN) on methylation data of hepatocellular cancer to identify tissue-specific differences with an *ACC* of 87% [16]. Similarly, Zeng et al. deployed CNN on 50 cancerous cell lines in combination with PCA to predict DNA methylation with 90% *ACC* [17]. In order to predict the methylation of the CpG site at an exact single base resolution, Tian et al. presented MRCNN, which made use of the correlation between DNA sequence patterns and their methylation state [18]. To obtain the expected value, the model employed fully linked layers, convolution, pooling, and one-hot encoding. By utilising a continuous loss function, it accomplished an apparent regression of methylation *ACC* of around 93.20%. For the purpose of methylation prediction, the 2D convolution method included additional DNA sequence features. Nevertheless, the predicted outcomes remained unchanged if the sequence was fixed. Furthermore, current DNA encoding methods, such as word embedding and one-hot encoding, gave priority to local information while ignoring global linkages.

In 2021, Fu et al. investigated the prediction of methylation states using a new combination of CNN and the Feed Forward Neural Network (FFNN) that integrated DNA sequence data with MeDIP-seq and histone modification data [19]. The model achieved an impressive AUROC of 0.977, outperforming traditional methods and indicating a substantial improvement in predicting DNA methylation states. It was found that the study concluded that the incorporation of epigenomic data alongside DNA sequence data significantly enhanced the *ACC* of DNA methylation state prediction. Wu et al. presented yet another combinatory method of CNN and Recurrent Neural Network (RNN) models. This fusion DL approach could predict DNA methylation status with an *ACC* of 84.90% [20]. Though RNNs excelled while extracting sequential characteristics, they struggled to uncover correlations between elements that were widely apart. Furthermore, Gomes et al. explored feature selection with DL to predict breast cancer by utilising methylation markers [21]. In their study, the authors demonstrated a high prediction accuracy of 98.75% using Illumina 450 K methylation data.

Several DL architectures have been capable of handling diverse medical image modalities and have attained high *ACC* in analysis and made diagnosis for a range of malignancies, such as cervical and breast cancer. The most commonly used diagnostic technique for the early detection of cervical cancer is the pap-smear test, but due to human error, it frequently yields false-positive results. Attallah et al. presented a computer-aided diagnostic model that integrated CNN for cervical cancer diagnosis [22]. The investigation improved the classification accuracy of pap-smear test images by up to 100%. It combined the spatial DL features with manually created descriptors from other domains, such as spatial and time-frequency domains. Similarly, Pacal et al. utilised DL techniques for the prediction of cervical cancer. It focused on the application of CNN and transformer approaches, along with ensemble learning, to improve *ACC* of cervical cancer classification using the pap-smear dataset [23,24]. Apart from using pap-smear tests, Ma et al. explored the development of a predictive model for cervical cancer utilising AI and data processing [25]. The research identified 34,389 differentially methylated CpG sites and constructed a model based on four CpG sites using Cox regression analysis, which demonstrated an AUROC of 0.833. However, Mallik et al. explored K-Nearest Neighbour (KNN) as a pre-processing technique along with FFNN in detecting DNA methylation and gene expression with an *ACC* of 90.69% [26]. The DNA sequence patterns are highly influenced by their neighbourhood information, but the application of FFNN-based models usually results in the loss of such information.

### 2.3. Problem Statement

Despite achieving high predictive accuracy in DNA methylation analysis, existing DL architectures remain largely confined to single-domain, standalone models, thereby limiting their ability to capture the complexity of epigenetic regulation in cervical carcinogenesis. In particular, the cervical cancer detection literature lacks hybrid frameworks that integrate complementary feature-selection strategies to simultaneously enhance predictive performance and biological interpretability. Conventional deep learning algorithms often provide abstract, high-dimensional features with limited biological significance, complicating the translation of computational predictions into clinically useful insights. Furthermore, current methodologies depend heavily on large, manually annotated datasets, which increase both the complexity and cost of medical evaluation. Sequential pattern analysis across multiple window sizes within epigenetic regions and promoter regions of tumour suppressor genes has not been fully explored, where robust parameterisation of DNA methylation patterns is essential for accurate modulation of gene expression. The statistical validation of methylation signatures across varying window sizes of DNA sequences is still limited, thereby reducing model generalisability and clinical relevance.

To address these gaps, this study proposes a hybrid DL-based framework that combines the UNet architecture with an advanced feature analysis technique, the Multi-Head Attention Network (MHAN). By analysing 100 DNA motifs within promoter regions, the framework aims to (i) enhance diagnostic accuracy for cervical cancer, (ii) improve the biological interpretability of extracted features, and (iii) predict statistically validated methylation signatures. This approach aims to advance epigenetic biomarker discovery and facilitate the future clinical deployment of methylation-based diagnostics.

**Table 1:** List of existing literature demonstrating the performance of various methods employed for predicting DNA methylation in cancer cells.

S. No.	Author	Dataset	Pre-Processing	Model	Accuracy/Sensitivity/Specificity/Matthews Correlation Coefficient/Area Under Receiver Operator Curve
1.	Liangrui Pan [27], 2023	Breast cancer (BRCA), glioblastoma (GBM), sarcoma (SARC), lung adenocarcinoma (LUAD), and stomach cancer (STAD) [28]	Multi-omics clustering method	Supervised Multi-Head Attention mechanism	100.00/-/-/-/-
2.	Rahul Gomes [21], 2022		Random forest, ANOVA	CNN	98.75/-/-/-/98.70
3.	Yue Ma [25], 2022	miRNA + mRNA datasets for cervical cancer [28]	Functional normalisation	Cox Proportional Hazard Regression Analysis model	-/-/-/-/83.30
4.	Changde Wu [20], 2022	GEO, GSE152204 [29]	CNN + RNN + one-hot encoding	ResNet	84.90/-/-/-/-
5.	Jing Tian [14], 2022	Gliomas [28]	Autoencoder + univariate Cox-pH models/ANOVA feature ranking	SVMs/K-means clustering	-/-/-/-/-
6.	Rocio del amor [15], 2022	Breast cancer [29]	Autoencoder	Autoencoder-based deep-embedded refined clustering model	99.27/-/-/-/-
7.	Saurav Mallik [26], 2020	Uterine cervical cancer dataset from NCBI [30,31]	Voom normalisation and Limma	FFNN	90.69/73.97/97.63/78.23/85.80
8.	Laiyi Fu [19], 2019	Cell lines: GM12878 and K562 [32]	Convolution layers + one-hot encoding		-/-/-/-/97.70
9.	Qi Tian [18], 2019	Non-cancerous: H1-ESC; cancerous: white matter of brain, lung tissue, and colon tissue [29]	One-hot encoding	CNN	>93.20/>95.00/85.00/-/96.00
10.	Haoyang Zeng [17], 2017	Fifty human cancer cell lines [32]			90.00/-/-/-/85.40
11.	Christof Angermueller [16], 2017	Hepatocellular cancer [29]			87.00/80.00/90.00/-/92.00

## 3. Methodology

**Figure 2:**
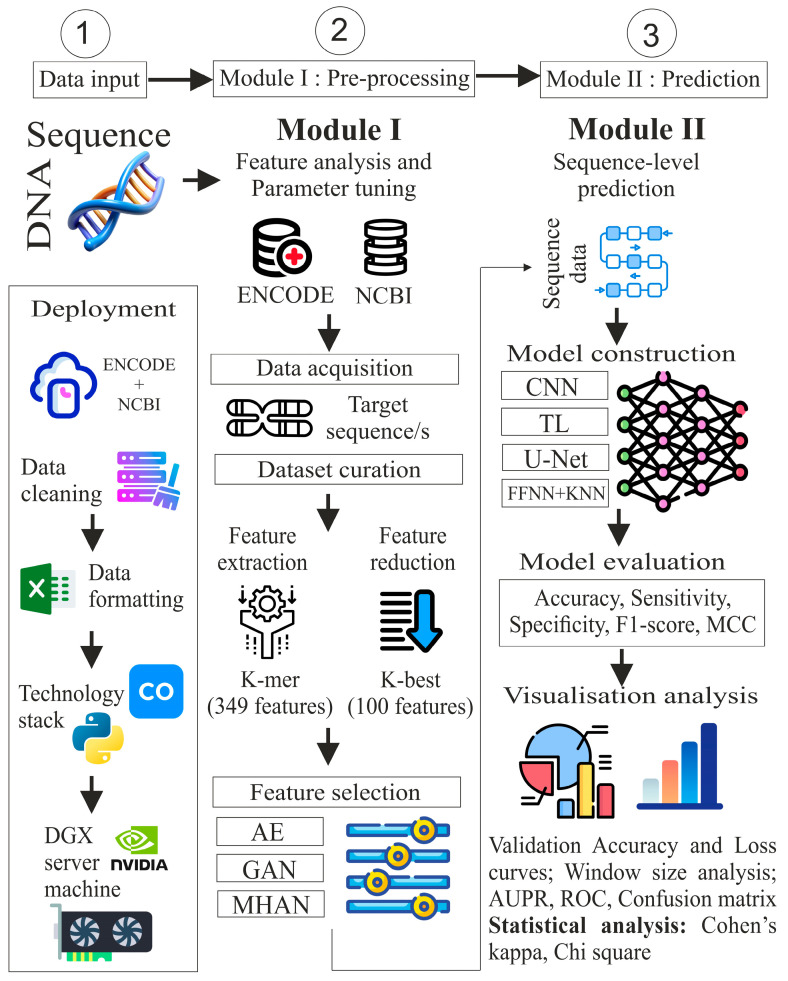
Proposed methodology for DNA methylation prediction in cervical cancer.

The proposed study includes five steps: data acquisition, dataset curation, feature extraction, feature selection, and classification. These steps have been categorised under two modules: pre-processing and prediction, as illustrated in Figure 2. In the pre-processing module, the data for ‘chromosome 4’ was acquired from two public benchmark repositories, ENCODE and NCBI. The DNA sequence was segmented and transformed into different sequence lengths in data curation. After this, the feature extraction sub-module uses a *k*-mer algorithm to extract local features. Extracted features were reduced using the K-best algorithm. Deep learning approaches, namely, autoencoders (AEs), Generative Adversarial Networks (GANs), and MHAN were applied for model-based feature selection. Furthermore, in the next module, the methylation status of CG sites was predicted in the uterine cervical cancer cell line through the intervention of CNN, FFNN-KNN, TL, and UNET. The performance of these models was evaluated using multiple window sizes through evaluation metrics, visualisation analysis, and statistical analysis.

The deployment module illustrates the pipeline execution upon the dataset curated in an MS-Excel spreadsheet by utilising Python version 3.6.9 in Google Collaboratory and the DGX server. The DGX server is a powerful device designed for high-performance computing that is specifically optimised for AI and DL workloads. The server has a processor of AMD EPYC 7742—64-Core along with a RAM of DDR4 1 TB 3200 MHz. It consists of 8 × 40 GB A100-SXM4 graphic processing units.

### 3.1. Data Acquisition

The study used two public databases, namely, ENCODE and the National Center for Biotechnological Information (NCBI), for the purpose of research reliability. High-throughput DNA methylation data from the bisulphite-sequenced dataset on cervical cancer cell line-HeLa was downloaded from ENCODE website [32]. The dataset contains chromosomal locations and the percent methylation value of 51,181 CG sites devised from experimental results. The second database, NCBI, holds a pivotal place in the domain of biotechnological sciences [33]. Thus, its platform was used to download data from normal human cells. The usage of both these databases helped to curate the dataset of this study.

### 3.2. Dataset Curation

HeLa is an immortalised cell line which consists of DNA methylation data for all 24 chromosomes. Thus, it was of great significance to select the chromosomes which could contribute to the generalisability of the algorithm. To avoid the extremes, chromosome 4 was chosen due to its median length range, among other factors. These chromosomal data, along with the methylation data, were used to build the dataset, which was carried out in two steps of data pre-processing as follows:

#### 3.2.1. Feature Extraction

**Figure 3:**
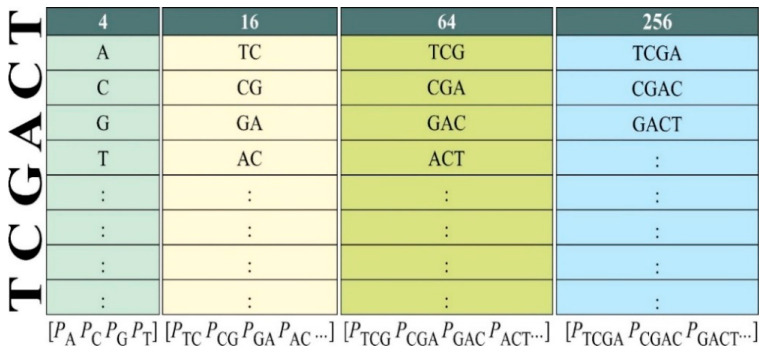
Mechanism for extraction of parameters using *k*-mer algorithm.

Figure 3 illustrates this step in which an algorithm *k*-mer based on permutation and combination was applied to extract DNA motifs using the four nucleotide bases: Adenine, Cytosine, Guanine, and Thymine [34]. Five types of *n*-mers were extracted using this algorithm, i.e., 4 monomers, 16 dimers, 64 trimers, 256 tetramers, along with only 9 different hexamers reported previously. The comprehensive extraction of lower-order *n*-mers (up to tetramers) is practicable and often utilised in DNA methylation research. The selection of hexamers is supported by previous biological research and statistical significance, demonstrating substantial correlation with DNA methylation, hence providing great discriminative capacity without overfitting the model with 4096 possible combinations [35,36]. This conforms to recognised methodologies of dimensionality reduction and motif prioritisation. Consequently, these nine hexamers function as biologically informed, high-impact features that improve model performance while reducing redundancy and computational load. Therefore, 349 motifs were taken into account through this method.

#### 3.2.2. Data Consolidation

After extracting motifs, the methylation values of each CG site were bifurcated into classes: methylated site and non-methylated site. The feature used to classify the methylation percent values is called Beta (β). For this research, CG sites with methylation percent values equivalent to or more than 50% were considered methylated or unmethylated as this cutoff score is better suited for prediction, as reported by previous studies [16,20,35,36,37,38,39,40]. Each CG site was then centred around a window size, which was set to 500 base pairs (bps). This window size delivered the maximal *ACC* in the proposed approach. The sequence of each site, in accordance with window size, was extracted from the GenBank accession no. CM000666.1 (GRCh 37.p13/hg19) human genomic dataset of the NCBI database [33]. Thereafter, the occurrence frequency of DNA motifs was counted in the 500 bp sequence around each CG site (±249 bp) as the final step of data consolidation.

### 3.3. Feature Analysis

Dataset consolidation was followed by feature reduction. For this, the K-best algorithm was used to minimise the number of features from 349 to just 100. The K-best technique involves a systematic expansion of the tree, starting at the root and progressing towards the leaves [41]. At each stage, this method identifies the most promising candidates by considering the lowest achievable path metric at each level. Hence, these 100 parameters can potentially exert a fundamental influence on DNA methylation, which may provide insights into other significant epigenetic alterations. This step is succeeded by the application of three different DL-based pre-processing techniques: autoencoders, Generative Adversarial Networks, and Multi-Head Attention Networks for the purpose of feature selection. The above-mentioned techniques have been chosen over other staple pre-processing techniques like PCA, LSTM, etc., because AE, MHAN, and GAN can capture complex, non-linear interactions in the data to model these complex patterns [42,43,44,45]. Moreover, MHAN can address the issues of long training times and difficulties in learning long-range dependencies with its attention mechanism and parallelisation capabilities [46,47]. Although these techniques have been used in several facets of epigenomic data of pap-smear test images of cervical cancer, it has been noted that they have not been fully explored as pre-processing algorithms in the domain of alphanumeric methylation data associated with cervical cancer [22,23,24,25,26]. Therefore, all three techniques have been applied separately in combination with multiple classification models. This approach yielded nine combinations of workflows for their thorough comparison of performance.

In the context of feature analysis, AE operates by compressing input information into a lower-dimensional depiction and then reconstructing the output to match the input. The hidden layer(s) of an AE is trained to extract the most important characteristics from the input data. By training the network to minimise reconstruction error, the algorithm learns to retain only the most salient features, effectively performing feature selection and extraction [48]. The encoder and decoder stages make up two primary stages of an autoencoder’s architecture. Using a non-linear mapping function, $Z=f_{\phi }(X)$, in which “ϕ” are trainable parameters of encoder architecture, the encoder step converts the input data “X” into a latent representation “Z”. To escape the curse of dimensionality, the dimensionality of latent space “Z” is significantly smaller than that of corresponding input information [49]. The most notable aspects of data can be represented by the latent space because it is a non-linear combination of the input data with reduced dimensionality. Reconstructing data using the parameters inherent in latent space, ${R=g}_{\theta }(Z)$ is the output of the decoder stage. It is necessary for the rebuilt representation “R” to resemble “X” as closely as is feasible. Consequently, an autoencoder model is optimised using the formula illustrated by Equation (1), given a set of data samples $X=\{x_{i},\ldots ,x_{n}\}$, where “n” is the total number of accessible samples:

(1) $$\underset{\theta ,\phi }{min}L_{rec}=\min\frac{1}{n}\sum_{i=1}^{n}{\left\|x_{i}-g_{\theta }\left(f_{\phi }\left(x_{i}\right)\right)\right\|}^{2}$$

Here, θ and ϕ stand for encoder and decoder’s parameters, respectively.

GAN has emerged as a powerful tool for feature extraction and selection in the field of ML. GANs are trained to produce representations that capture the essential characteristics of input data, facilitating dimensionality reduction and feature learning. Simultaneously, for feature selection, GAN can help identify the most relevant features by focusing on those that are crucial for generating data close to the real distribution. This dual capability makes GAN a versatile pre-processing technique, enhancing model performance by focusing on salient features and mitigating the impact of irrelevant or noisy data. Due to improvements made to the architecture by research in recent years, the GAN architecture has experienced several advancements [50]. Goodfellow et al. proposed that a generator “G” and a discriminator “D” make up the fundamental structure of GAN. The generator “G” generates new data points from a vector of input noise “z” represented as $P_{z}(z)$, and discriminator “D” categorises those data points as real or fake [51]. Thus, the ultimate objective of “G” is generating data points that are more realistic so that “D” is unable to recognise them as fraudulent. Every iteration of the network comprises a backpropagation phase wherein parameters of “G” are refined to yield more realistic-looking generated data points. Using the loss function in Equation (2), “G” and “D” are engaged in a minmax play [51].

(2) $${min}_{G}{max}_{D}(G,D)=[E_{x\sim P_{data}}[logD(x)]+E_{z\sim P_{z}(z)}[log(1–D(G(z)))]$$

More specifically, when “x” originates from a distribution of actual data denoted by $P_{data}(x)$, the discriminator aims to minimise the chance D(G(z)) that the data point is real and maximise the likelihood D(x) that “x” is real when G(z) is the generated data.

Apart from these two techniques, MHAN, as a pre-processing technique for feature extraction and selection, involves leveraging the attention mechanism, which is instrumental in identifying the importance of various parts of the input data. It employs multiple ‘heads’ of attention, allowing the model to concurrently focus on various subsections of input data and capture a wider range of dependencies. This parallel attention leads to a more comprehensive understanding of the input parameters, enhancing the model’s ability to distinguish as well as prioritise the most relevant features for a given task.

The position encoding module, a mechanism module for attention, a feature forward propagation module, and a perceptron module make up the majority of the MHAN mechanism encoder [52]. Initially, the position encoding module creates tiny feature tokens and cls tokens by linearly mapping the matrix to features as follows (Equation (3)):

(3) feature=input embedding+positional encoding

Adding position information to each location in the input sequence is known as position encoding, and it often involves combining the sine and cosine functions. For feature extraction, the feature linear mapping is thereafter incorporated into the MHAN mechanism’s encoder. The query, key, and value feature spaces are copied with features, and each is divided into several heads for processing. For every attention head, the similarity between “Q” and “K” is determined and normalised to a probability distribution [53]. It operates in the following manner (Equation (4)):

(4) $$Attention(Q_{i},K_{i},V_{i})=softmax(Q_{i}*K_{i})∕\mathrm{sqrt}(d_{k})*{\;V}_{i}$$

The Softmax function, where “K” is a feature dimension of $d_{k}$, normalises the similarity score to a probability distribution. Next, using a linear transformation, the output from each attention head is split, projected, and integrated as follows (Equation (5)):

(5) $$MultiHead=Concatenate(Attention(Q_{i},K_{i},V_{i}))*\;W$$

Here, the term ‘concatenate’ refers to splitting the output of each attention head, hence representing learning feature weight.

### 3.4. Proposed Model and Its Evaluation

Once the relevant features are selected, they are split into training and testing sets of 70:30. In this experiment, 35,287 methylation patterns of CGs were fed into a neural net-based classification model for training and tested on an unknown dataset of 15,354 CG sites. The construction of the model was undertaken with meticulous attention to architecture and parameter tuning to optimise the classification and prediction of DNA methylation patterns. UNet, originally designed for biomedical image segmentation, was adapted for this task due to its powerful architecture, which consists of a contracting path to capture context and a symmetric expanding path that enables precise localisation. The key elements of the UNet architecture are encoders, decoders, a bridge network, skip connections, a loss function criterion, and the process of binary conversion, sometimes known as the Softmax layer.

**Figure 4:**
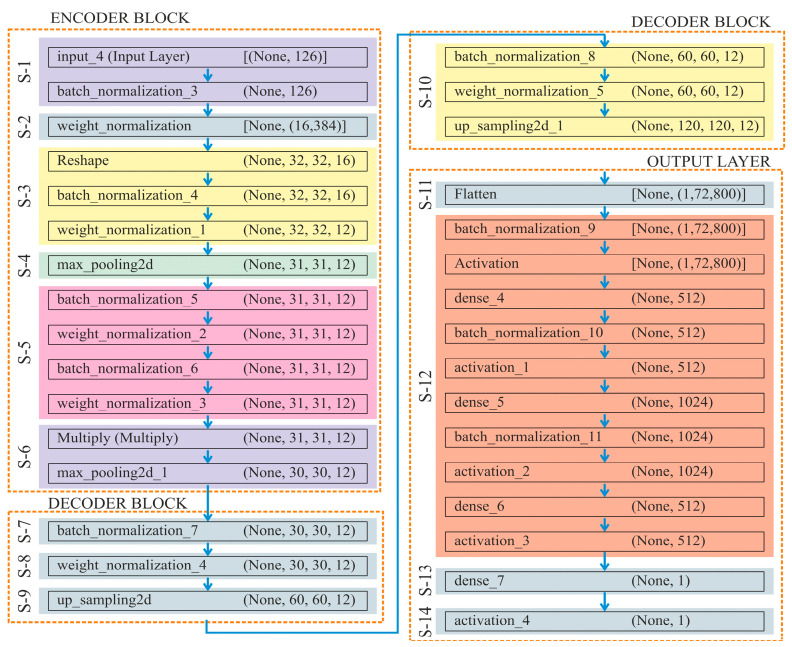
Architecture of the proposed classification model used to predict DNA methylation.

The incorporation of down-convolution and up-convolution, along with the capability to select the most significant information or features at the highest level, known as max pooling, greatly advances the process of automated feature extraction [54]. Moreover, this DL model effectively preserves the intended features during shape reconstruction (decoder phase) by transferring parameter information from the encoder to decoder phases. Thus, this structure is particularly beneficial for a detailed analysis of methylation patterns, allowing the model to focus on relevant characteristics at different scales while providing a precise prediction of methylation status. The detailed architecture of this algorithm has been described in Figure 4, which consists of 14 steps, where “S-1” refers to “Step-1”.

The incorporation of GAN or MHAN into this model introduced additional layers of complexity and refinement. GAN, with its generative and discriminative components, was used to augment the dataset and generate synthetic methylation profiles, thus providing a richer dataset for model training. On the other hand, MHAN, with its multi-headed attention mechanism, allowed models to focus on various parts of input sequences simultaneously, leading to a better understanding and prediction of methylation patterns.


**Variables**
  C: Channels  H: Height  M: Methylated  U: Unmethylated  W: Width  Input: X as $Tensor\left(H,W,C\right)$,  Output: Classification labels $F_{o}=M,U$.***Encoder*** ***Block***  ***Step 1:***    X1←Batch Normalisation (X)  ***Step 2:***    X2←Dense(X1,weights,biases)  ***Step 3:***    $R=Reshape\;(X2,\;\left(l,m,p\right))$    $R^{\prime }=Batch\;Normalisation\;\left(R\right)$    $X3\leftarrow Conv\;(R^{\prime },weights,biases)$  ***Step 4:***    X3←Apply Maxpooling on X3 for reducing spatial dimensions    $T_{skipconn}=X3$  ***Step 5:***    ${X3}^{\prime }=Batch\;Normalisation\;\left(X3\right)$    $X4\leftarrow Conv\;\left({X3}^{\prime },weights,biases\right)$    ${X4}^{\prime }=Batch\;Normalisation\;\left(X4\right)$    $X5\leftarrow Conv\;({X4}^{\prime },weights,biases)$  ***Step 6:***    Perform skip connection multiplication $X6=X5\times T_{skipconn}$    Y←Apply Maxpooling on X6 for reducing spatial dimensions
***Decoder Block***


for i=0 to 1



Start for

  ***Step 7:***    $Y1=Batch\;Normalisation\;\left(Y\right)$  ***Step 8:***    ${Y1}^{\prime }\leftarrow Conv\;(Y1,weights,biases)$  ***Step 9:***    
Increase spatial dimensions through Upsampling2D  ***Step 10:***    $Tensor\;\left(2H,2W,C\right)\leftarrow Tensor\;\left(H,W,C\right)$    $Z=Tensor\;\left(2H,2W,C\right)$

End For


***Output Layer***
  ***Step 11:***    Z1←Flatten(Z)  ***Step 12:***    $Z2\leftarrow ReLU(Batch\;Normalisation\;\left(Z1\right))$    $Z3=ReLU\left(Batch\;Normalisation\left(Dense\left(Z2,weights,biases\right)\right)\right)$    $Z4=ReLU\left(Batch\;Normalisation\left(Dense\left(Z3,weights,biases\right)\right)\right)$  ***Step 13:***    Apply a final dense layer to produce the number of labels    O=Dense(Z4,weights,biases)  ***Step 14:***    Apply a sigmoid activation to produce the final output    $F_{o}=Sigmoid(O)$

Overall, UNet has been divided into three sub-components: encoder, decoder, and output layer. In the **Encoder** block, (i) it normalises the input data to have a mean of 0 and a variance of 1, which helps in stabilising and speeding up the training process; (ii) it improves the training stability by normalising the weights of the dense layer. For this, a dense (fully connected) layer with 16,384 units has been applied to the normalised input; (iii) the reshape function transforms the dense layer into a tensor of shape 32×32×16. Here, 1D output of the dense layer is transformed into a 3D tensor, which is suitable for applying convolution operations. After reshaping, the tensor has been normalised, which further helps in the training of dataset. Furthermore, a 2D convolutional layer with 12 filters and a kernel size of 4, followed by ‘swish’ activation, has been applied; (iv) the spatial dimensions of the input tensor have been reduced by taking the maximum value over each 2×2 window, with a stride value of 1. Store the current state of the tensor “X” in the variable “X3”. This step saves intermediate tensor “X” for its later use in the skip connection; (v) as a single set, apply the BatchNormalisation() and WeightNormalisation() twice or repeatedly; (vi) multiply layer performs element-wise multiplication of the input tensors. Here, the current tensor “X6” gets multiplied elementwise with the saved tensor “X3”. Overall, this helps in retaining features from the encoder part. By using these layers and operations, the encoder compresses the input data into a lower-dimensional representation, capturing essential features while reducing spatial dimensions.

The **Decoder** block functions in the following manner:

(i) Here, the tensor “X” has been normalised by adjusting and scaling the activations; (ii) to improve training stability, a 2D convolutional layer with 12 filters, a kernel size of 4, ‘swish’ activation, and weight normalisation, has been used in the function WeightNormalisation(); (iii) **size = (2, 2)** in the function UpSampling2D(), which up-samples the tensor by a factor of 2 along both spatial dimensions (height and width); (iv) repeat steps (i)–(iii).

By applying these steps, the decoder module reconstructs the spatial dimensions of the tensor while retaining and refining the features extracted by the encoder.

The output layer processes learned features from the decoder and generates the final prediction as follows:

(i) Flatten layer here converts the multi-dimensional tensor output of the previous layer into a 1D vector, which makes it suitable for fully connected dense layers; (ii) a set of three functions, namely, BatchNormalisation(), Activation(), and Dense(), have been applied thrice; taking ‘*ReLU*’ as an activation function to add non-linearity and taking neurons count as 512, 1024, and 512 at 1st, 2nd and 3rd execution; (iii) Dense(num_labels) function adds final dense layer with num_labels units, corresponding to a number of output labels; (iv) finally, the output is squashed by the sigmoid activation function to a range of 0 to 1, which makes it appropriate for problems involving binary or multi-label classification.

By integrating these advanced neural network architectures and techniques, the study aimed to leverage their respective strengths in a complementary manner, ultimately enhancing the *ACC* and reliability of DNA methylation classification and prediction. The performance of the proposed model was analysed on the basis of the following indices: Equations (6)–(11) were calculated based on test results, such as the quantity of True Negative (*TN*), False Negative (*FN*), True Positive (*TP*), and False Positive (*FP*) samples concluded from the confusion matrix. This clear and structured approach provided additional information about the classifier, which was computed by projecting model outputs to expected (true) results.

(6) $$ACC=\frac{TP+TN}{TP+TN+FP+FN}$$

(7) $$SE=\frac{TP}{TP+FN}$$

(8) $$SP=\frac{TN}{TN+FP}$$

(9) $$MCC=\frac{TP.TN-FP.FN}{\sqrt{\left(TP+FP\right)(TP+FN)(TN+FP)(TN+FN)}}$$

(10) $$Precision=\frac{TP}{TP+FP}$$

(11) $$F1\;Score=\frac{TP}{TP+\frac{1}{\;2}(FP+FN)}$$

A DL model uses a loss function, which calculates the difference between expected and actual values, to learn how to map input data to corresponding target outputs throughout the training phase. Therefore, for visualisation analysis, learning curves, such as Validation–Accuracy (VA) curves, Validation–Loss (VL) curves, Area under Precision Recall Curves (AUPRC), and AUROC have been employed. The percentage of correctly identified cases in the new dataset is measured by the VA curve. On the other hand, the VL curve quantifies the model’s ability to generalise to a testing dataset. It shows the errors on updated data. To provide a comprehensive comparison of the performance of different models, the precision–recall scores and the AUROC scores are the basis for the calculation of the AUPRC curve and the AUROC, respectively. The AUROC graph indicates the likelihood that a randomly chosen authentic target will be ranked higher than a randomly chosen fake target. The ratio of *TP* to *FP* is shown on the AUROC graph. When evaluating a model’s performance on unbalanced datasets, the PR curve is commonly employed. In the same manner, the average of the interpolated precisions, AUPRC, can be calculated. Greater values of AUROC and AUPRC indicate better prediction performance for the underlying model.

### 3.5. Validation of Gene Promoter Region Methylation

**Figure 5:**
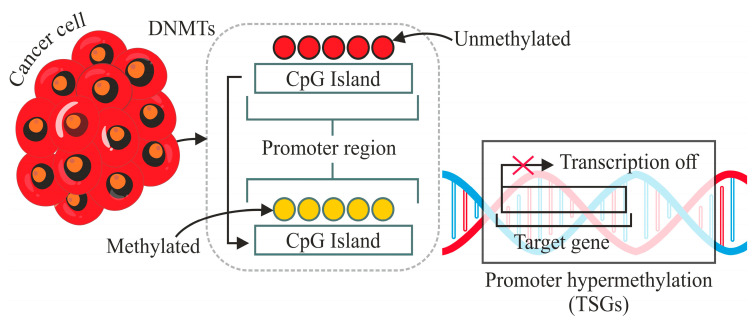
Role of DNA methylation in the control of gene expression through promoter region methylation of a gene.

It is well known that aberrant promoter hypermethylation, which silences genes, plays a crucial role in the development and spread of cancer. The minimum DNA region that controls the proper commencement of transcription is commonly referred to as the core promoter [55]. Thus, core promoter elements are a crucial component in the control of gene expression as the core promoter sequence consists of the Transcription Start Site (TSS) [56]. Therefore, DNA methylation in promoter regions of a gene may play a significant role in its dysregulation, as illustrated in Figure 5 [57]. Moreover, the prediction of methylation in gene promoter regions can also help in understanding the gene expression regulated via DNA methylation.

The proposed model has been validated by using the methylation status of promoter regions of five genes, namely, *miR-100*, *miR-138*, *miR-484*, *hTERT*, and *ERVH48-1*. Five studies have reported the association of promoter region methylation of these genes with cervical cancer cell lines [58,59,60,61,62]. After analysing the bisulphite sequencing data from the earlier reported experimental expression analysis and RT-PCR of these genes, sequences of both forward and reverse primers were used to map and extract CG sites from the human genomic assembly of NCBI. One hundred and twenty-eight CG sites in the promoter of the above-mentioned five genes were considered to determine their methylation values in the HeLa cells. This methylation data were obtained experimentally and published in the earlier reported studies [58,59,60,61,62]. These data were then converted into a binary dataset using the earlier stated β value. The dataset was denoised from outliers before validation, leaving out a final dataset of a total of 87 CG sites. This was followed by extracting the methylation pattern of about 500 bp centred around each of these sites. The proposed model was then applied to this new dataset for further evaluation to demonstrate considerable robustness. A statistical analysis based on both observed and predicted values was performed to show the model’s significant reliability.

## 4. Results and Discussion

### 4.1. Influence of Feature Selection Techniques

**Figure 6:**
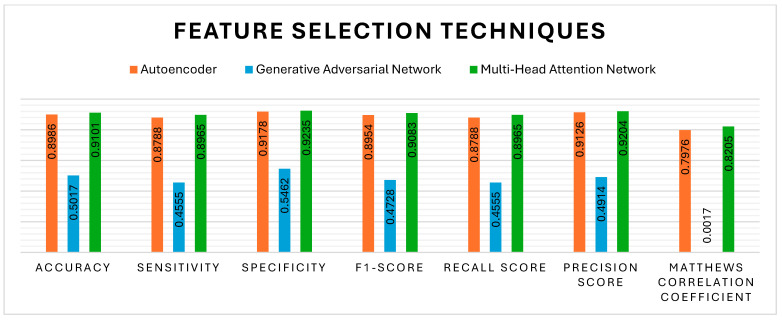
Performance evaluation of different feature selection methods with respect to DNA methylation prediction.

Apart from the K-best, the feature selection techniques, such as AE, GAN, and MHAN, have also significantly affected the methylation detection findings. All three pre-processing techniques were incorporated and evaluated separately to assess the robustness of the model. Figure 6 evidently shows that MHAN has outperformed the AE in terms of evaluation metrics, whereas the performance of GAN was poor, indicating its inability to capture important features in association with the classification model in relation to the methylation pattern.

**Figure 7:**
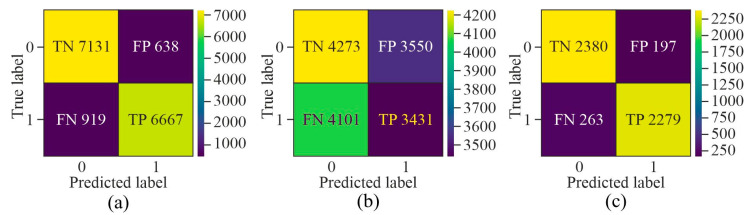
Confusion matrix for the proposed model using different feature selection techniques on the HeLa cell line—(**a**) AE, (**b**) GAN, and (**c**) MHAN.

While evaluating the confusion matrix derived from the three distinct selection techniques, as shown in Figure 7, it can be observed that upon applying GAN as a pre-processing method, the number of samples labelled as *FN* and *FP* was very high as compared to *TN* and *TP*. Although AE showed the highest number of *TP* and *TN* among all three methods, it labelled more than 500 samples as *FN* and *FP*. On the other hand, MHAN, in combination with UNet, showed the least number of samples labelled as *FP* and *FN*, which indicated that it selected features with more association with methylation patterns, leading to a more accurate classification of methylated and unmethylated CpGs.

**Figure 8:**
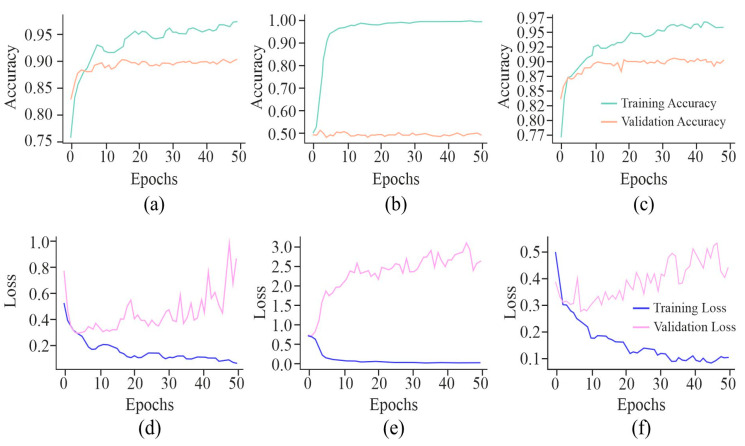
Learning curves of the proposed model using different feature selection techniques on the HeLa cell line with a 500 bp window size represented by VA curve of AE, GAN, and MHAN as (**a**), (**b**), and (**c**), respectively; and VL curve of AE, GAN, and MHAN as (**d**), (**e**), and (**f**), respectively.

It can be observed from the learning curves, as shown in Figure 8, that VA curves of UNet in combination with either AE or MHAN did not display much difference between the performance of the model on training and testing data. It proved the model’s ability to apply what it had learned to new data. However, GAN coupled with UNet showed some contradictory results, indicating the model’s overfitting issue. On the other hand, an increasing validation loss due to the use of GAN with UNet made it less effective while generalising to unseen datasets. Furthermore, AE with UNet exhibited frequent fluctuations during the final stage of validation, indicating the issues related to the model or data, such as a high learning rate causing instability, etc. The VL curve of UNet using MHAN as a feature selector algorithm showed a slight increase in validation loss and less training loss, suggesting that the model had effectively learned to fit the training data.

**Figure 9:**
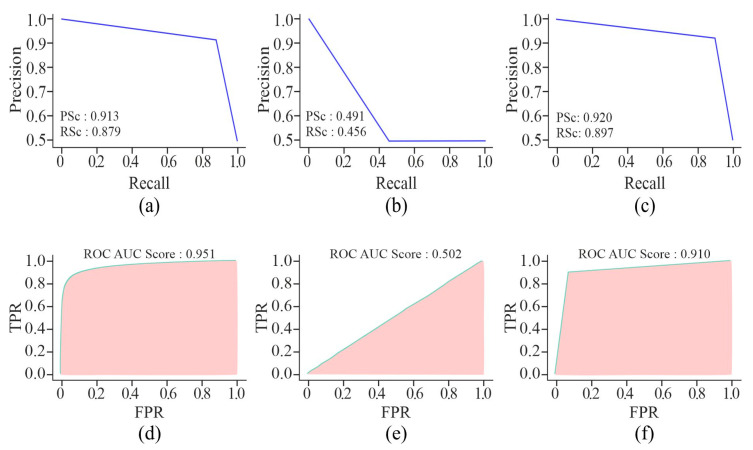
Visualisation curves of the proposed model using different feature selection techniques on the HeLa cell line with a 500 bp window size represented by AUPRCs of AE, GAN, and MHAN as (**a**), (**b**) and (**c**), respectively; and AUROCs of AE, GAN, and MHAN as (**d**), (**e**), and (**f**), respectively.

By using AE or MHAN with UNet, the precision and recall scores were almost similar in the case of AUPRCs, though higher in MHAN, as shown in Figure 9. However, the combination of GAN and UNet yielded scores below 0.500, indicating a huge difference in actual and predicted methylation. The AUROC scores of AE and MHAN reflected their better performance by exhibiting high sensitivity (*SE*). However, UNet, along with GAN, failed to discriminate between positive and negative samples. This was indicated by the formation of a diagonal line between *TP* and *FP* rates, suggesting a no-skill model.

### 4.2. Influence of Window Size Variation

**Figure 10:**
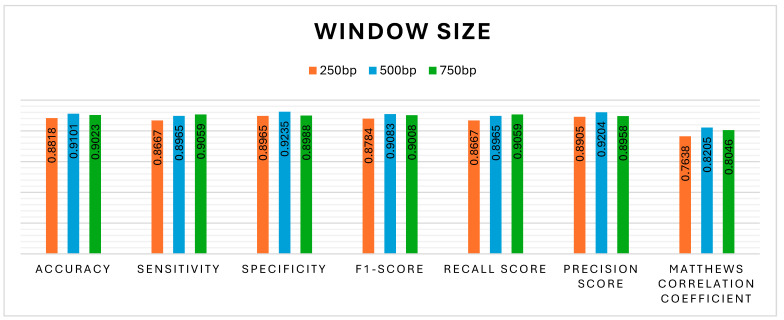
Influence of sequence length on DNA methylation prediction.

**Figure 11:**
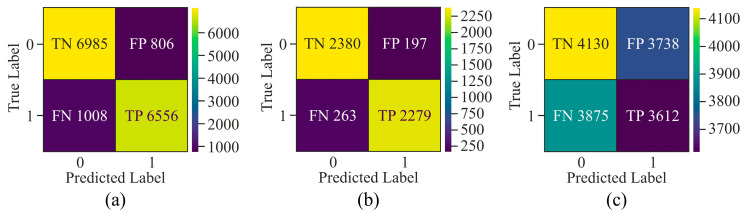
Confusion matrix of the proposed model on HeLa cell line with different window sizes—(**a**) 250 bp; (**b**) 500 bp; and (**c**) 750 bp.

**Figure 12:**
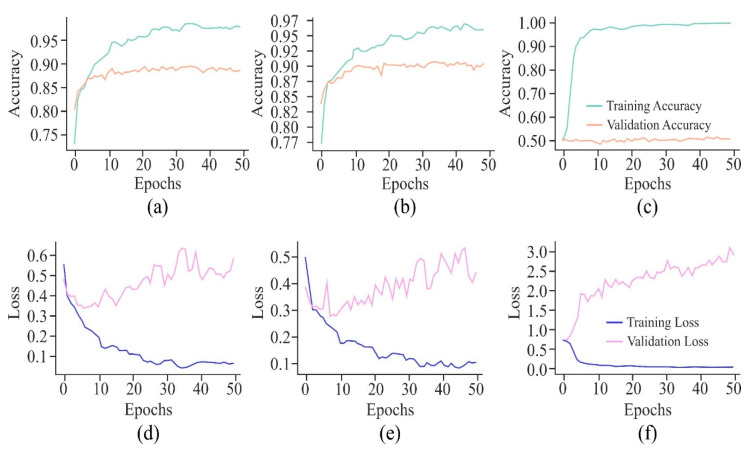
Learning curves of the proposed model on the HeLa cell line with different window sizes represented by the VA curve of 250, 500, and 750 bp as (**a**), (**b**), and (**c**), respectively; and VL curve of 250, 500, and 750 bp as (**d**), (**e**), and (**f**), respectively.

**Figure 13:**
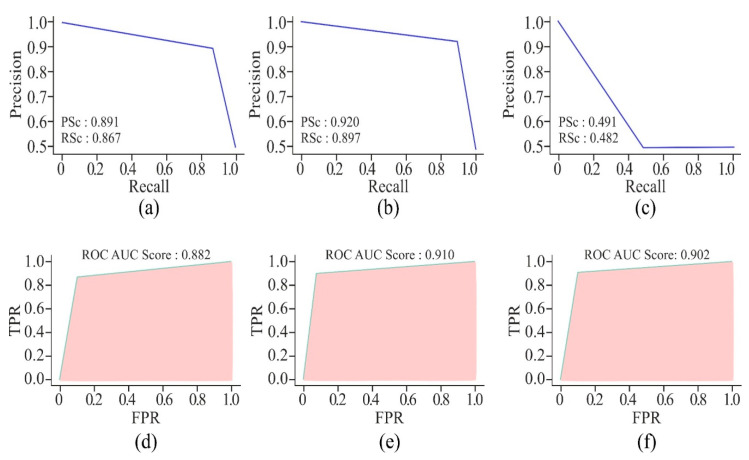
Visualisation curves of the proposed model on the HeLa cell line with different window sizes represented by AUPRCs of 250, 500, and 750 bp as (**a**), (**b**), and (**c**), respectively; and AUROCs of 250, 500, and 750 bp as (**d**), (**e**), and (**f**), respectively.

The proposed model investigated three different lengths of DNA sequence, i.e., 250, 500, and 750 bp. However, the most accurate results were produced by the 500 bp length of the DNA sequential base pairs: A, C, G, and T, as shown in Figure 10. The observation that a 500 bp window yields the best performance in methylation prediction is indeed consistent with findings in existing research. Specifically, the work of Takai and Jones [63,64] has demonstrated that CpG island definitions based on regions ≥ 500 bp in length, with GC content ≥ 55% and an observed-to-expected CpG ratio ≥ 0.65, are more likely to correspond to gene promoter regions and are less likely to include repetitive elements such as Alus. This refined criterion enhances the biological relevance of CpG island detection and has become a preferred standard for studies involving methylation prediction. Moreover, the proposed model’s preference for the 500 bp window is consistent with empirical findings, as this length effectively captures meaningful methylation patterns while reducing noise from non-functional CpG-like sequences. The enhanced performance at this window size likely stems from its alignment with biologically validated CpG island parameters, which are enriched in regulatory features sensitive to methylation. Thus, the findings have been in agreement with the CpG island criteria proposed by Takai and Jones, reinforcing the appropriateness of the 500 bp window size for accurate and biologically meaningful methylation prediction. The results pertaining to the UNet-MHAN model are highlighted in Figure 10, Figure 11, Figure 12 and Figure 13, whereas the experimental results with 250 and 750 bp are shown below.

It can be observed from the confusion matrix highlighted in Figure 11 that window sizes of 250 bp and 750 bp had larger samples labelled as *FN* and *FP* as compared to the confusion matrix derived from experimentation on the 500 bp window size. Moreover, a comparison between the window sizes of 250 bp and 750 bp showed that *TN* and *TP* in 250 bp were greater than those of 750 bp. Apart from this, the model exhibited data imbalance during the 250 bp window size analysis as compared to the 750 bp window size.

The VA curves of 250 and 500 bp input length, as displayed in Figure 12, reflected that the validation *ACC* in both the curves was almost similar, but the training *ACC* was greater in the case of the 500 bp window size, indicating greater influence of this window size on the proposed model. However, with a window size of 750 bp, validation *ACC* was stagnant at its lowest value. In the case of VL curves of all these three window sizes, a huge difference existed between the performances of the proposed model on training and testing data with window sizes of 250 bp and 750 bp, raising the issue of overfitting of the model. On the other hand, with an input length of 500 bp, the model showed improvement in its performance due to high loss in the initial training, which decreased gradually as the loss reflected the errors between actual data and predicted values.

Figure 13 highlights that the AUPRC of 750 bp is less than 0.500, which is a poor performance. However, the model with a window size of 250 bp has shown better prediction of DNA methylation, with the scores ranging between 0.860 and 0.891. These scores are still less than those produced by the model with a window size of 500 bp. On the other hand, the AUROC scores of all three models with different window sizes are almost the same, but the model with 500 bp DNA sequence length recorded the highest score, which proved it to be a perfect skill model.

### 4.3. Comparison with State-of-the-Art Models

#### 4.3.1. Convolutional Neural Network

As the proposed model is based on CNN, thus, CNN as a standalone model has been chosen to compare the classification of DNA methylation with the performance of UNet. Features selected through AE, GAN, and MHAN were incorporated into CNN one by one for methylation prediction in the form of different workflows. Usually, the CNN model excels in capturing local patterns through its convolutional layers. This can be particularly useful in identifying specific methylation signatures, as the convolutional filters could effectively detect spatial hierarchies and patterns within the genomic sequences. In CNN, the convolutional layer convolves the features from the previous layer using trainable kernels and activation functions, resulting in feature maps as the output [65]. As a result, different input features are combined with each feature map output. If there are exactly “n” output mappings in the CNN’s pooling layer, then there must be “n” input maps as well [66]. Not only do convolution and pooling functions perform their usual roles in CNN, but they also contribute to non-linearity. Equation (12) demonstrates the convolution operation employed in the convolutional layer:

(12) F(i,j)=(I∗K)(i,j)=∑∑I(i+m, j+n)K(m,n)

where “I” is for the input matrix; “K” represents a 2D filter of size m×n; and “F” stands for the output of a 2D feature map. The convolutional layer representation is with “I∗K”. Therefore, by employing various kernel sizes and depths, the CNN model can offer a robust framework for subsequent classification tasks.

#### 4.3.2. Feed Forward Neural Network with K-Nearest Neighbour

This hybrid model has been structured using FFNN and KNN in a successive manner for methylation classification. In FFNN, neurons are organised sequentially, where each neuron computes a weighted sum of its respective inputs. Input and output neurons serve to transmit and receive external signals [67]. Neurons that are not exposed to the external environment but are connected internally are termed ‘hidden neurons’. These hidden neurons function as intermediaries, effectively bridging the external inputs with the network outputs. The inclusion of one or more hidden layers enables the model to capture complex statistical features [68]. FFNNs are fully connected and non-recurrent, operating without loops. Mathematically, this algorithm can be expressed as a function given in Equation (13), which maps network inputs $x_{i}$ to target outputs $y_{k}$ [69]. This equation corresponds to a network containing a single hidden layer. The weights $w_{ij}$ define connections from inputs to the hidden layer, where the index i→j indicates summation over these links. Bias terms for hidden units are represented as $\alpha_{j}$, and the activation functions at hidden nodes are denoted as $f_{j}$. Connections from the hidden layer to the output layer are indexed by j and k.

(13) $$y_{k}=f_{k}\left(\alpha_{k}+\sum_{j\to k}W_{jk}f_{j}\left(\alpha_{j}+\sum_{i\to j}W_{ij}x_{i}\right)\right)$$

This algorithm, in combination with KNN has been used to develop the final model. The extracted hidden layer output of FFNN for all data points has been used as a feature for KNN. The nearest neighbour algorithm identifies the K  closest data points to an unknown instance within an *n*-dimensional space $R^{n}$, and infers its class based on these K neighbours [70]. These neighbours are termed the K-nearest neighbours of the target sample. The method assumes that each instance is a point in $R^{n}$, and proximity is determined using Euclidean distance [71]. Equation (14) displays the feature vector of instance x as follows:

(14) $$\left\langle a_{1}(x),a_{2}(x),...,a_{n}(x)\right\rangle$$

where $a_{r}(x)$ denotes the *r*-th attribute of x. The distance between two instances $x_{i}$ and $x_{j}$ is given by Equation (15):

(15) $$d\left(x_{i}x_{j}\right)=\sqrt{\sum_{r=1}^{n}\left(a_{r}\left(x_{i}\right)-a_{r}\left(x_{j}\right)\right)2}$$

In this context, nearest neighbour learning defines a classification function ${f:\;R}^{n}->V$, where $V=\{v_{1},v_{2},\;\ldots \;v_{s}\}$ is a finite set of classes. The choice of K depends on both the quantity and distribution of samples within each class and can vary across different applications.

#### 4.3.3. Transfer Learning

Transfer learning is a technique of machine learning which involves adapting a model developed for one task to another but the related one. Furthermore, a pre-trained model is essentially an existing model that has evolved into a DL standard. Thus, the application of these models provides greater confidence about their effective working. In this study, ResNet has been used as a pre-trained model with a CNN network. It employs an identity function to cope with the exploding/vanishing gradient issue. The concept of residual blocks serves as the foundation for the ResNet architecture. The network makes use of “skip connections”, a method that creates connections by eschewing particular intermediate layers between the activations of one layer and those of a subsequent layer. A residual block is created as a result of this operation [72]. Several residual blocks are stacked to create ResNet. ResNet enables layers to learn residual functions with reference to the layer inputs rather than requiring each layer to explicitly learn the intended underlying mapping. This facilitates the learning of identity mappings as required, which aids the network in more efficiently training deeper designs. Rather than denoting “$H\left(x\right)$” for the initial mapping in Equation (16), it is proposed that the network be allowed to adjust and align with data in the following manner [73]:

(16) $$F\left(x\right)≔H\left(x\right)-x$$

which gives $H\left(x\right)≔\;F\left(x\right)+x$, where “x” refers to identity; $H\left(x\right)$ = initial mapping; and $F\left(x\right)$ = network fitting.

**Figure 14:**
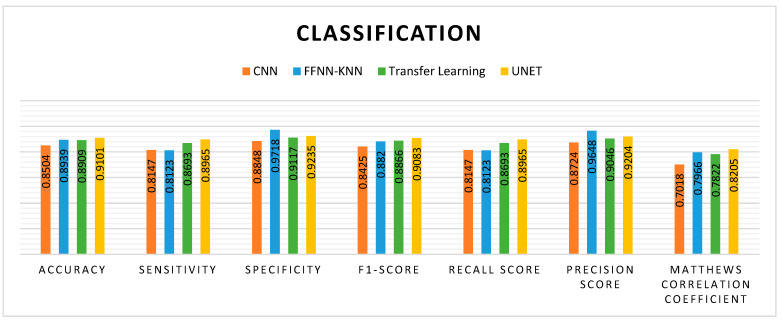
Performance comparison of different models with UNet with respect to DNA methylation prediction.

The comparative performance of four models, including CNN, FFNN-KNN, ResNet, and UNet, in combination with MHAN as a pre-processing technique in predicting the methylation status of a CG site in a sample, is presented in Figure 14.

**Figure 15:**
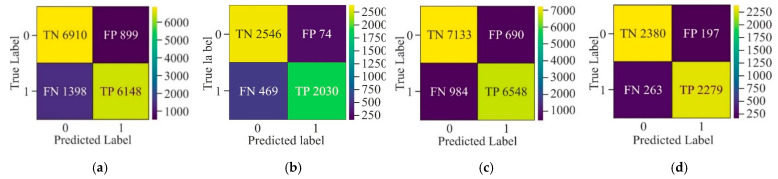
Confusion matrix of various models on HeLa with MHAN as a feature selection technique on a window size of 500 bp—(**a**) CNN, (**b**) FFNN-KNN, (**c**) Transfer Learning, and (**d**) UNet.

Figure 15 clearly shows that CNN has attained a high number of *TP* and *TN*, which means that it has correctly identified positive and negative cases. However, certain *FP* and *FN* still exist, which indicates that there is room for improvement in *ACC*. Moreover, FFNN-KNN demonstrates a balanced classification with a relatively low number of *FP* (74) and *FN* (449) and a substantial number of *TP* (2030) and *TN* (2546). This suggests that this hybrid model performs reasonably well in reducing misclassification errors while maintaining a stable detection performance. On the other hand, the Transfer Learning model has a balanced but less reliable performance due to higher misclassification rates. Furthermore, the UNet model has appeared as the most efficient due to its remarkable precision in minimising *FP* and *FN*. Its ability to maintain low misclassification rates makes it particularly suitable for scenarios where the cost of *FP* and *FN* is as high as medical diagnostics.

**Figure 16:**
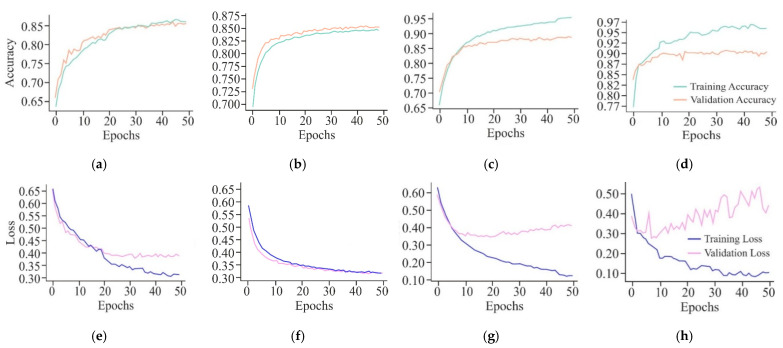
Learning curves of different models on HeLa with MHAN as a feature selection technique on a window size of 500 bp represented by the VA curve of CNN, FFNN-KNN, Transfer Learning, and UNet as (**a**), (**b**), (**c**), and (**d**), respectively; and VL curve of CNN, FFNN-KNN, Transfer Learning, and UNet as (**e**), (**f**), (**g**), and (**h**), respectively.

The VA curves shown in Figure 16 indicate that the CNN model has emerged as a good fit for the training data and generalises well to the validation data, indicating that it has learned to recognise patterns in the cancerous cell line data effectively without significant overfitting. The FFNN-KNN model exhibits a smooth convergence with high and closely aligned training and validation accuracy curves, reaching values around 0.87. This reflects the model’s balanced learning capability and its effectiveness in minimising overfitting. On the other hand, the initial high *ACC* of ResNet indicates that the model has benefited significantly from the pre-trained weights, which quickly adapt to the cancerous cell line data. However, there is a small gap between training and validation *ACC*, suggesting minor overfitting. Moreover, UNet model shows a high training *ACC* and maintains a slightly higher validation *ACC* around 0.90. The gap between training and validation *ACC* is minimal, indicating that the model effectively generalises the validation data. This is expected of a model designed for segmentation tasks, where precise localisation and context understanding are crucial. Apart from this, when the CNN shows a consistent decrease in training and validation loss, the validation loss plateau indicates that the model has reached its learning capacity and might not improve further without additional data or architectural change. Moreover, the FFNN-KNN model displays a smooth and steady decline in training and validation loss curves, converging near 0.30. This suggests that the model has learned effectively from the data and generalises well, showing minimal signs of overfitting and strong learning stability throughout the training process.

Furthermore, ResNet has shown rapid learning and a significant reduction in training loss, but the plateauing of validation loss at a relatively higher level indicates potential overfitting. This suggests that the model may have learned too much from the training data, making it less effective on unseen data. Although the UNet shows variability in validation loss, it demonstrates a strong learning capability with a significantly reduced training loss. The variability in validation loss might be attributed to the model’s sensitivity to the complex patterns in the data, which could be advantageous in identifying subtle features in cancerous cell line data.

**Figure 17:**
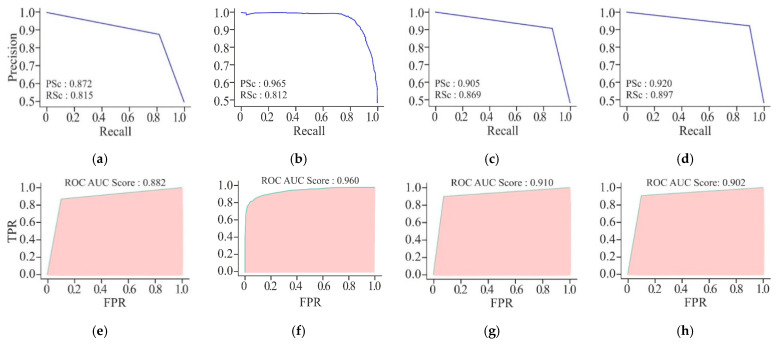
Visualisation curves of different models on HeLa with MHAN as a feature selection technique on a window size of 500 bp represented by AUPRCs of CNN, FFNN-KNN, Transfer Learning, and UNet as (**a**), (**b**), (**c**), and (**d**), respectively; and AUROCs of CNN, FFNN-KNN, Transfer Learning, and UNet as (**e**), (**f**), (**g**), and (**h**), respectively.

Figure 17 displays the precision–recall curve of CNN model. The precision starts at a high value but drops significantly as the recall increases. It indicates that the CNN model performs well while identifying *TP* initially, but its performance is adversely affected when it attempts to recall more instances. The FFNN-KNN model presents a consistently high precision across almost the entire range of recall, suggesting that the hybrid model effectively maintains high precision while achieving strong recall with minimal trade-off between the two metrics. The curve of ResNet remains higher for a longer range of recall values, demonstrating that the ResNet model maintains better precision while recalling more instances. Of the three models, the UNet model has exhibited the highest precision and recall scores. The precision–recall curve for UNet is consistently high, showing a minimal drop in precision even if the recall increases. Overall, the UNet model has maintained a good balance between precision and recall, making it the most reliable model for accurately identifying cancerous cell lines. On the other hand, the AUROC curve for the CNN model has shown a relatively good performance with an AUROC score of 0.916. This indicates that the CNN model is quite effective in distinguishing between positive and negative instances. In the case of FFNN-KNN, a strong AUROC performance has been demonstrated with a score of 0.960. Its curve follows a steep trajectory, reflecting a high true positive rate at a low false positive rate. The ResNet model performs better than the CNN model, with a higher AUROC score of 0.946. The AUROC is closer to the top-left corner, indicating better discrimination between positive and negative instances. This higher score of AUROC suggests that the ResNet model can classify cancerous and non-cancerous cells more correctly than the CNN model. In the context of cancer detection, the ability to minimise *FN*, i.e., high recall and ensure high precision, is paramount. Thus, despite a slightly lower AUROC score, the performance of UNet in these metrics is the greatest of all the models.

### 4.4. Validation on Promoter Region Methylation

The methylation status of the promoter regions of five genes—*miR-100*, *miR-138*, *miR-484*, *hTERT*, and *ERVH48-1*—was also used to validate the proposed framework, as mentioned earlier. All five genes had different methylation patterns in their promoter region, constituting 87 CG positions around the TSS, which were methylated and unmethylated in association with cervical cancer, as reported earlier. Therefore, the proposed model has been validated on these CG positions, demonstrating its robustness by achieving an *ACC* of 81.60% in methylation prediction in the promoter regions of the aforementioned genes. Moreover, the F1-score, which balances precision and recall, is relatively high, indicating the robust performance of the model.

**Figure 18:**
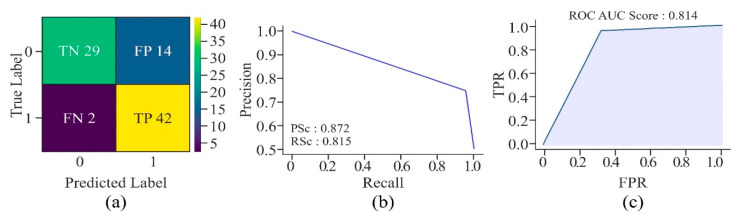
Performance of the proposed model in relation to methylation prediction on multiple CG sites present in promoter regions of various genes associated with cervical cancer—(**a**) confusion matrix, (**b**) AUPRC, and (**c**) AUROC.

The confusion matrix, as shown in Figure 18, describes the robustness of the UNet model in accurately identifying promoter regions of cervical cancer-causing genes. The overall accuracy of the model shows that it has performed well on the given dataset. Although the precision score is moderately high, which indicates the existence of some *FP*, a good recall score proves the model’s strength in identifying almost all the *TP*, which is crucial in medical applications, where missing a true case, i.e., *FN* may lead to significant consequences. The PR curve’s shape, showing high precision and recall for the majority of thresholds, further establishes the model’s robustness and reliability for this task. In addition, with an AUROC of 0.814, the model has demonstrated good discriminatory power to effectively differentiate between *TP* and *TN*. The model has also shown a balanced performance in terms of sensitivity and specificity. Thus, it is reliable for practical applications, particularly in the context of identifying crucial genomic regions associated with cervical cancer.

### 4.5. Statistical Analysis

The integration of robust statistical methods with DL approaches can significantly contribute towards understanding and developing predictive capabilities in the field of DNA methylation and beyond. Thus, the model has used evaluation metrics to predict methylation values for a sample of 87 CG sites through statistical analysis. Several statistical techniques are used for the study of DNA methylation data. However, the conflicting results derived from the same dataset make it difficult to select the most effective statistical method. Hence, keeping in view the type of data, the chi-square test has been applied on this dataset to determine the association between two categorical variables [74]. In this case, the association between observed and predicted values of methylation and non-methylation has been tested.

(17) $$\chi^{2}=\sum \frac{{\left(O_{ij}-E_{ij}\right)}^{2}}{E_{ij}}$$

(18) $$\mathrm{d}f=\left(\mathrm{rows}-1\right)\times \left(\mathrm{columns}-1\right)$$

(19) $$p\text{-}value=1-F\left(\chi^{2},\mathrm{d}f\right)$$

Here, $F\left(\chi^{2},\mathrm{d}f\right)$ is the cumulative distribution function of the chi-square distribution with Degrees of Freedom (ⅆf).

The calculated chi-square statistic “$\left(\chi^{2}\right)$” is 34.82 as per Equation (17), which reflects how much the observed frequencies deviate from the expected frequencies under the assumption of independence. This significant chi-square test result has led to establishing a strong association between the observed methylation states and the predictions made by the UNet model. The model’s predictions are not due to any random chance but show a meaningful pattern in relation to the observed values. Furthermore, the *p*-*value* in a chi-square test has also been calculated using the “$\chi^{2}$” statistic and “ⅆf” through Equations (17)–(19). The *p*-value is the probability of observing a chi-square statistic as extreme as, or more than extreme, the calculated “$\chi^{2}$” value, given that the null hypothesis is true [75]. The resultant *p*-value of $3.62\times 10^{-19}$ is extremely low, indicating a highly significant result. Apart from this, Cohen’s Kappa (κ) has also been calculated for these data, which is a statistical measure of inter-rater agreement for categorical items [76]. It is generally considered to be a more robust measure than a simple percent agreement calculation because “κ” takes into account the possibility of the agreement occurring by chance. The value of “κ” is calculated using the Equation (20):

(20) $$\kappa =\frac{P_{o}-P_{e}}{1-P_{e}}$$

(21) $$P_{o\;}=\frac{TP+TN}{TP+TN+FP+FN}$$

(22) $$P_{e}=\left(\frac{TP+FP}{TP+TN+FP+FN}\times \frac{TP+FN}{TP+TN+FP+FN}\right)+\left(\frac{TN+FN}{TP+TN+FP+FN}\times \frac{TN+FP}{TP+TN+FP+FN}\right)$$

where

“$P_{o}$” is the relative observed agreement among raters, i.e., *ACC*, calculated through Equation (21).
“$P_{e}$” is the hypothetical probability of chance agreement calculated using the observed data with the help of Equation (22).

The “κ” value of 0.631, as derived from the above equations, indicates a substantial agreement between the observed and predicted values. It shows that the UNet model’s predictions are significantly better than those expected by chance alone. This value suggests that the model is quite reliable in predicting methylation and unmethylation status.

### 4.6. Generality on Data

**Figure 19:**
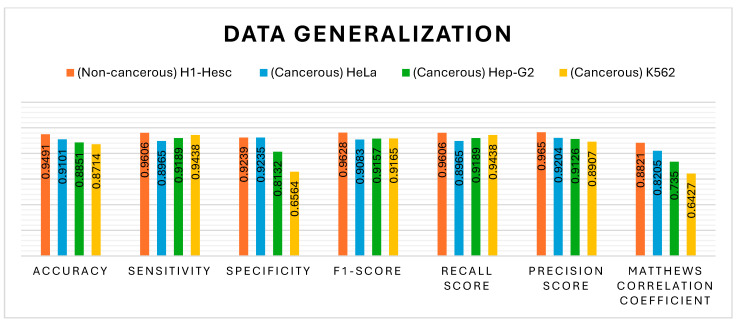
Performance comparison with other cell lines.

**Figure 20:**
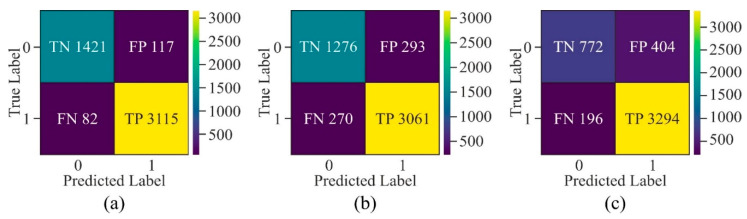
Confusion matrix of the proposed model with window size of 500 bp upon different cell lines—(**a**) H1-hESC, (**b**) HepG2, and (**c**) K562.

**Figure 21:**
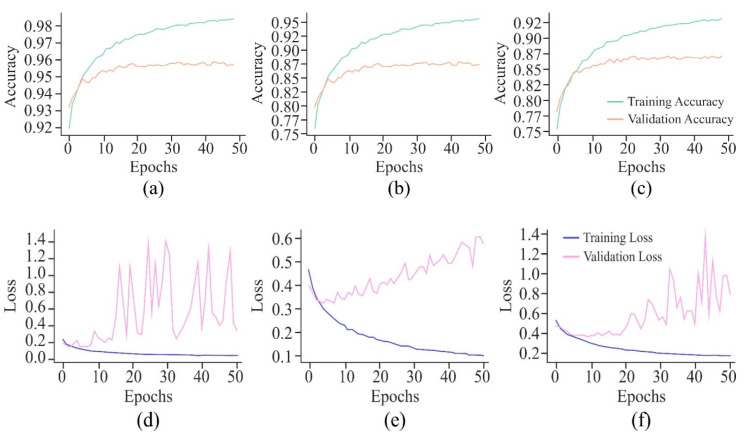
Learning curves of the proposed model with a window size of 500 bp upon different cell lines represented by VA curve of H1-hESC, HepG2, and K562 as (**a**), (**b**), and (**c**), respectively; and VL curve of H1-hESC, HepG2, and K562 as (**d**), (**e**), and (**f**), respectively.

**Figure 22:**
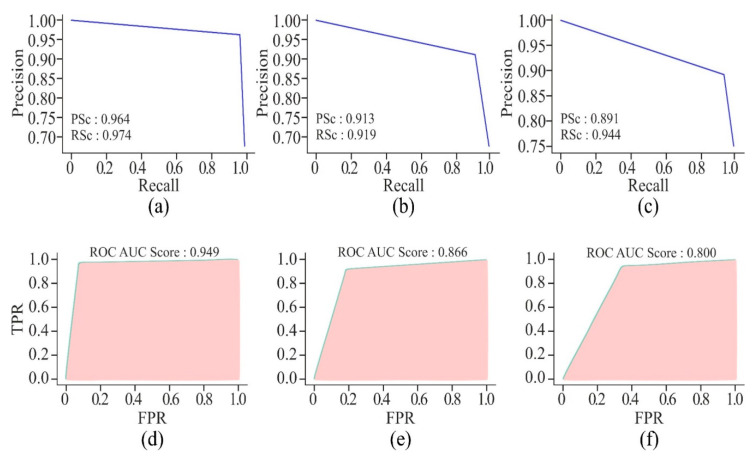
Visualisation curves of the proposed model with a window size of 500 bp upon different cell lines represented by AUPRCs of H1-hESC, HepG2, and K562 as (**a**), (**b**), and (**c**), respectively; and AUROCs of H1-hESC, HepG2, and K562 as (**d**), (**e**), and (**f**), respectively.

The generalisation ability of the proposed model has also been tested by extending its application to other types of cell lines: two cancerous and one non-cancerous, as illustrated in Figure 19. The non-cancerous cell line H1-hESC and the cancerous cell lines HepG2 and K562 are constituted of 47,346, 48,996, and 46,659 CG positions, respectively, along with their methylation value. The proposed model has been tested with UNet as a classification model along with MHAN, as shown in Figure 19, Figure 20, Figure 21 and Figure 22 using different evaluation methods. This demonstrates that the proposed model has the potential to be applied to different DNA methylation data.

The confusion matrix of the non-cancerous cell line, as shown in Figure 20, has a lesser number of *FN* and *FP* as compared to cancerous cell lines, indicating that the model learned the patterns accurately to generalise on testing data. Although the samples labelled as *FN* and *FP* in all three cancerous cell lines have almost the same values, the values of true negatives are very few in the K562 cell line. Thus, the highest *ACC* has been obtained in the HeLa cell line in all three cancerous cell lines.

The VA curve, as displayed in Figure 21, reflects that the training *ACC* is higher than the validation *ACC* in all the cases, indicating that the model fits well in the training data. However, the validation *ACC* is consistently lower for cancer cell lines in comparison to the non-cancer cell line, indicating that it is more challenging to predict DNA methylation in cancer cell lines. The consistent gap between training and validation *ACC* in all cancer cell lines suggests that the model overfits the training data. This overfitting is higher in cancer cell lines, implying that the model might be sensitive to specific features in the training set, leading to inconsistent performance on new data, most probably due to the complexity and variability of DNA methylation patterns in cancer cells. In the case of VL curves, it can be observed from Figure 21 that the training loss has decreased steadily in all the cell lines, indicating effective learning from the training data. However, in the non-cancerous cell line, the validation loss has shown significant fluctuations, and it stabilises at a higher value, indicating the challenges in the generalisation of unseen data, suggesting sensitivity towards specific features in the training set and inability to capture the underlying patterns necessary for generalisation. Moreover, in HeLa and HepG2 cell lines, it can be observed from Figure 8 and Figure 21 that a fluctuation pattern exists at the beginning of the VL curve, which stabilises later around 0.5, with some peaks indicating the least overfitting. In the K562 cell line, the model has captured noise and specific details in the training data that do not translate well to the validation set, as indicated by the fluctuations.

It can be seen from Figure 22 that the non-cancer cell line has achieved the greatest precision and recall, suggesting that it is somewhat easier to predict DNA methylation in non-cancerous cells. The curves illustrate the trade-off between precision and recall. The closeness of all the curves to the top-right corner is a sign of good performance. The cancer cell lines, HepG2 and K562, have shown a slight drop in precision as compared to HeLa, suggesting the scope for improvement in reducing false positives. In addition, all the cell lines have shown relatively high AUROC scores, indicating that the model has performed well in distinguishing between positive and negative classes. Among the cancer cell lines, HeLa has recorded the highest AUROC score, followed by the scores of HepG2 and K562. This decrease in scores indicates greater difficulty in accurately predicting the DNA methylation patterns in the cancer cell lines.

## 5. Conclusions, Limitations, and Future Work

Methylation identification for DNA sequences is a crucial task in the field of epigenetics. It may be used as a biomarker for cancer diagnosis, a target for therapeutic interventions, and a criterion for prognostic evaluation. Conventional techniques for methylation detection depend more on time- and money-consuming wet experiments. However, DL has made methylation detection easier these days through signal processing. It is still a matter of great concern to increase the robustness and *ACC* of state-of-the-art techniques. This research work has explored four DL models that include CNN, FFNN-KNN, Transfer Learning, and UNet in combination with an advanced feature selection method known as MHAN. The combination of UNet-MHAN has emerged as the most suitable choice for the prediction of DNA methylation in cervical cancer cell line as it achieved an accuracy level of 91.01% along with sensitivity of 89.65%, specificity of around 92.35% and an area under curve of 0.910. In addition, the model was tested on five genes showcasing the significance of methylation prediction in their promoter region with an accuracy of 81.60% and an area under curve score of 0.814 along with a *p*-value of $3.62\times 10^{-19}$, and Cohen’s Kappa value of 0.631 from further statistical analysis. However, the study’s real-world relevance can be improved through the imperative process of further validation in varied clinical situations. These limitations, in any manner, do not diminish the strength of the current research work, rather, the suggestions made here are meant to improve and broaden the applicability of the proposed model in future.

## Data Availability

The dataset/s that support the findings of this study are openly available at University of California, Santa Cruz (UCSC), Genomics Institute [URL (accessed on 22 May 2025): https://genome.ucsc.edu/cgi-bin/hgFileUi?db=hg19&g=wgEncodeHaibMethylRrbs], and National Library of Medicine—National Center for Biotechnology Information (NCBI) [URL (accessed on 22 May 2025): https://www.ncbi.nlm.nih.gov/nuccore/NC_000004.11/].

## References

[1] Smith Z.D., Meissner A. (2013). DNA methylation: Roles in mammalian development. Nat. Rev. Genet..

[2] Handa V., Jeltsch A. (2005). Profound flanking sequence preference of Dnmt3a and Dnmt3b mammalian DNA methyltransferases shape the human epigenome. J. Mol. Biol..

[3] Jeltsch A., Jurkowska R.Z. (2014). New concepts in DNA methylation. Trends Biochem. Sci..

[4] Gardiner-Garden M., Frommer M. (1987). CpG islands in vertebrate genomes. J. Mol. Biol..

[5] Clarke M.A., Wentzensen N., Mirabello L., Ghosh A., Wacholder S., Harari A., Lorincz A., Schiffman M., Burk R.D. (2012). Human papillomavirus DNA methylation as a potential biomarker for cervical cancer. Cancer Epidemiol. Biomark. Prev..

[6] Lu Q., Ma D., Zhao S. (2012). DNA methylation changes in cervical cancers. Cancer Epigenetics: Methods and Protocols.

[7] Choi J., Chae H. (2020). methCancer-gen: A DNA methylome dataset generator for user-specified cancer type based on conditional variational autoencoder. BMC Bioinform..

[8] Hashem E.M., Kamal A., Mabrouk M.S., Fakhre M.W. (2024). Predicting DNA Methylation state of CpG Islands Using Machine Learning. J. Adv. Eng. Trends.

[9] Troyanskaya O., Trajanoski Z., Carpenter A., Thrun S., Razavian N., Oliver N. (2020). Artificial intelligence and cancer. Nat. Cancer.

[10] Fang F., Fan S., Zhang X., Zhang M.Q. (2006). Predicting methylation status of CpG islands in the human brain. Bioinformatics.

[11] Previti C., Harari O., Zwir I., del Val C. (2009). Profile analysis and prediction of tissue-specific CpG island methylation classes. BMC Bioinform..

[12] Jiang L., Wang C., Tang J., Guo F. (2019). LightCpG: A multi-view CpG sites detection on single-cell whole genome sequence data. BMC Genom..

[13] Bedon L., Dal Bo M., Mossenta M., Busato D., Toffoli G., Polano M. (2021). A novel epigenetic machine learning model to define risk of progression for hepatocellular carcinoma patients. Int. J. Mol. Sci..

[14] Tian J., Zhu M., Ren Z., Zhao Q., Wang P., He C.K., Zhang M., Peng X., Wu B., Feng R. (2022). Deep learning algorithm reveals two prognostic subtypes in patients with gliomas. BMC Bioinform..

[15] Amor R.D., Colomer A., Monteagudo C., Naranjo V. (2022). A deep embedded refined clustering approach for breast cancer distinction based on DNA methylation. Neural Comput. Appl..

[16] Angermueller C., Lee H.J., Reik W., Stegle O. (2017). DeepCpG: Accurate prediction of single-cell DNA methylation states using deep learning. Genome Biol..

[17] Zeng H., Gifford D.K. (2017). Predicting the impact of non-coding variants on DNA methylation. Nucleic Acids Res..

[18] Tian Q., Zou J., Tang J., Fang Y., Yu Z., Fan S. (2019). MRCNN: A deep learning model for regression of genome-wide DNA methylation. BMC Genom..

[19] Fu L., Peng Q., Chai L. (2019). Predicting DNA methylation states with hybrid information based deep-learning model. IEEE/ACM Trans. Comput. Biol. Bioinform..

[20] Wu C., Yang H., Li J., Geng F., Bai J., Liu C., Kao W. Prediction of DNA methylation site status based on fusion deep learning algorithm. Proceedings of the 5th International Conference on Advanced Electronic Materials, Computers and Software Engineering (AEMCSE).

[21] Gomes R., Paul N., He N., Huber A.F., Jansen R.J. (2022). Application of feature selection and deep learning for cancer prediction using DNA methylation markers. Genes.

[22] Attallah O. (2023). Cervical cancer diagnosis based on multi-domain features using deep learning enhanced by handcrafted descriptors. Appl. Sci..

[23] Pacal I., Kılıcarslan S. (2023). Deep learning-based approaches for robust classification of cervical cancer. Neural Comput. Appl..

[24] Pacal I. (2024). MaxCerVixT: A novel lightweight vision transformer-based Approach for precise cervical cancer detection. Knowl.-Based Syst..

[25] Ma Y., Zhu H., Yang Z., Wang D. (2022). Optimizing the Prognostic Model of Cervical Cancer Based on Artificial Intelligence Algorithm and Data Mining Technology. Wirel. Commun. Mob. Comput..

[26] Mallik S., Seth S., Bhadra T., Zhao Z. (2020). A linear regression and deep learning approach for detecting reliable genetic alterations in cancer using DNA methylation and gene expression data. Genes.

[27] Pan L., Qin P., Rong P., Zeng X., Liu D., Peng S. PACS: Prediction and analysis of cancer subtypes from multi-omics data based on a multi-head attention mechanism model. Proceedings of the 2023 IEEE International Conference on Bioinformatics and Biomedicine (BIBM).

[28] (2005). The Cancer Genome Atlas Project. http://cancergenome.nih.gov/.

[29] Barrett T., Wilhite S.E., Ledoux P., Evangelista C., Kim I.F., Tomashevsky M., Marshall K.A., Phillippy K.H., Sherman P.M., Holko M. (2012). NCBI GEO: Archive for functional genomics data sets—Update. Nucleic Acids Res..

[30] Zhuang J., Jones A., Lee S.-H., Ng E., Fiegl H., Zikan M., Cibula D., Sargent A., Salvesen H.B., Jacobs I.J. (2012). The dynamics and prognostic potential of DNA methylation changes at stem cell gene loci in women’s cancer. PLoS Genet..

[31] Teschendorff A.E., Jones A., Widschwendter M. (2016). Stochastic epigenetic outliers can define field defects in cancer. BMC Bioinform..

[32] Davis C.A., Hitz B.C., Sloan C.A., Chan E.T., Davidson J.M., Gabdank I., Hilton J.A., Jain K., Baymuradov U.K., Narayanan A.K. (2018). The Encyclopedia of DNA elements (ENCODE): Data portal update. Nucleic Acids Res..

[33] (2004). Gene (Internet).

[34] Wong K.C., Chan T.M., Peng C., Li Y., Zhang Z. (2013). DNA motif elucidation using belief propagation. Nucleic Acids Res..

[35] Khwaja M., Kalofonou M., Toumazou C. A deep belief network system for prediction of DNA methylation. Proceedings of the 2017 IEEE Biomedical Circuits and Systems Conference (BioCAS).

[36] Wrzodek C., Büchel F., Hinselmann G., Eichner J., Mittag F., Zell A. (2012). Linking the epigenome to the genome: Correlation of different features to DNA methylation of CpG islands. PLoS ONE.

[37] Pedregosa F. (2017). Scikit-learn: Machine learning in python Fabian. J. Mach. Learn. Res..

[38] Zheng H., Wu H., Li J., Jiang S.W. (2013). CpGIMethPred: Computational model for predicting methylation status of CpG islands in human genome. BMC Med. Genom..

[39] Pavlovic M., Ray P., Pavlovic K., Kotamarti A., Chen M., Zhang M.Q. (2017). DIRECTION: A machine learning framework for predicting and characterizing DNA methylation and hydroxymethylation in mammalian genomes. Bioinformatics.

[40] Zou L.S., Erdos M.R., Taylor D.L., Chines P.S., Varshney A., Parker S.C.J., Collins F.S., Didion J.P., The McDonnell Genome Institute (2018). BoostMe accurately predicts DNA methylation values in whole-genome bisulfite sequencing of multiple human tissues. BMC Genom..

[41] Lawler E.L. (1972). A procedure for computing the k best solutions to discrete optimization problems and its application to the shortest path problem. Manag. Sci..

[42] Shahbazi M.A. (2022). Developing Artificial Intelligence Tools to Investigate the Phenotypes and Correlates of Chronic Kidney Disease Patients in West Virginia. Master’s Thesis.

[43] Shaikh T.A., Rasool T., Lone F.R. (2022). Towards leveraging the role of machine learning and artificial intelligence in precision agriculture and smart farming. Comput. Electron. Agric..

[44] Camacho J., Picó J., Ferrer A. (2010). Data understanding with PCA: Structural and variance information plots. Chemom. Intell. Lab. Syst..

[45] Arslan E., Schulz J., Rai K. (2021). Machine learning in epigenomics: Insights into cancer biology and medicine. Biochim. Biophys. Acta (BBA)-Rev. Cancer.

[46] Bukhsh Z.A., Saeed A., Dijkman R.M. (2021). Processtransformer: Predictive business process monitoring with transformer network. arXiv.

[47] Hassanin M., Anwar S., Radwan I., Khan F.S., Mian A. (2024). Visual attention methods in deep learning: An in-depth survey. Inf. Fusion.

[48] Min E., Guo X., Liu Q., Zhang G., Cui J., Long J. (2018). A survey of clustering with deep learning: From the perspective of network architecture. IEEE Access.

[49] Xie J., Girshick R., Farhadi A. (2016). Unsupervised deep embedding for clustering analysis. Proceedings of the International Conference on Machine Learning.

[50] Wang Z., She Q., Ward T.E. (2021). Generative adversarial networks in computer vision: A survey and taxonomy. ACM Comput. Surv. (CSUR).

[51] Goodfellow I., Pouget-Abadie J., Mirza M., Xu B., Warde-Farley D., Ozair S., Courville A., Bengio Y. (2014). Generative adversarial nets. Adv. Neural Inf. Process. Syst..

[52] Han K., Wang Y., Chen H., Chen X., Guo J., Liu Z., Tang Y., Xiao A., Xu C., Xu Y. (2022). A survey on vision transformer. IEEE Trans. Pattern Anal. Mach. Intell..

[53] Pan L., Wang H., Wang L., Ji B., Liu M., Chongcheawchamnan M., Yuan J., Peng S. (2022). Noise-reducing attention cross fusion learning transformer for histological image classification of osteosarcoma. Biomed. Signal Process. Control..

[54] Ronneberger O., Fischer P., Brox T. (2015). U-net: Convolutional networks for biomedical image segmentation. Proceedings of the Medical Image Computing and Computer-Assisted Intervention–MICCAI 2015: 18th International Conference.

[55] Butler J.E., Kadonaga J.T. (2002). The RNA polymerase II core promoter: A key component in the regulation of gene expression. Genes Dev..

[56] Kadonaga J.T. (2012). Perspectives on the RNA polymerase II core promoter. Wiley Interdiscip. Rev. Dev. Biol..

[57] Kulis M., Esteller M. (2010). DNA methylation and cancer. Adv. Genet..

[58] Renaud S., Loukinov D., Abdullaev Z., Guilleret I., Bosman F.T., Lobanenkov V., Benhattar J. (2007). Dual role of DNA methylation inside and outside of CTCF-binding regions in the transcriptional regulation of the telomerase hTERT gene. Nucleic Acids Res..

[59] Hu Y., Wu F., Liu Y., Zhao Q., Tang H. (2019). DNMT1 recruited by EZH2-mediated silencing of miR-484 contributes to the malignancy of cervical cancer cells through MMP14 and HNF1A. Clin. Epigenet..

[60] Yao T., Yao Y., Chen Z., Peng Y., Zhong G., Huang C., Li J., Li R. (2022). CircCASC15-miR-100-mTOR may influence the cervical cancer radioresistance. Cancer Cell Int..

[61] Chen R., Gan Q., Zhao S., Zhang D., Wang S., Yao L., Yuan M., Cheng J. (2022). DNA methylation of miR-138 regulates cell proliferation and EMT in cervical cancer by targeting EZH2. BMC Cancer.

[62] Sugimoto J., Schust D.J., Sugimoto M., Jinno Y., Kudo Y. (2023). Controlling Trophoblast Cell Fusion in the Human Placenta—Transcriptional Regulation of Suppressyn, an Endogenous Inhibitor of Syncytin-1. Biomolecules.

[63] Takai D., Jones P.A. (2002). Comprehensive analysis of CpG islands in human chromosomes 21 and 22. Proc. Natl. Acad. Sci. USA.

[64] Takai D., Jones P.A. (2003). The CpG island searcher: A new WWW resource. Silico Biol..

[65] Muhammad L.J., Algehyne E.A., Usman S.S. (2020). Predictive supervised machine learning models for diabetes mellitus. SN Comput. Sci..

[66] Algehyne E.A., Jibril M.L., Algehainy N.A., Alamri O.A., Alzahrani A.K. (2022). Fuzzy neural network expert system with an improved Gini index random forest-based feature importance measure algorithm for early diagnosis of breast cancer in Saudi Arabia. Big Data Cogn. Comput..

[67] Bebis G., Georgiopoulos M. (1994). Feed-forward neural networks. IEEE Potentials.

[68] Haykin S. (1994). Neural Networks: A Comprehensive Foundation.

[69] Ripley B.D. (1996). Neural Network Discriminant Analysis: Statistical Aspects.

[70] Yu Z., Chen H., Liu J., You J., Leung H., Han G. (2015). Hybrid $ k $-nearest neighbor classifier. IEEE Trans. Cybern..

[71] Wang L. (2019). Research and implementation of machine learning classifier based on KNN. IOP Conference Series: Materials Science and Engineering.

[72] Targ S., Almeida D., Lyman K. (2016). Resnet in resnet: Generalizing residual architectures. arXiv.

[73] Residual Networks (ResNet)—Deep Learning. https://www.geeksforgeeks.org/residual-networks-resnet-deep-learning/.

[74] Tallarida R.J., Murray R.B., Tallarida R.J., Murray R.B. (1987). Chi-square test. Manual of Pharmacologic Calculations: With Computer Programs.

[75] Rana R., Singhal R. (2015). Chi-square test and its application in hypothesis testing. J. Prim. Care Spec..

[76] Blackman N.J.M., Koval J.J. (2000). Interval estimation for Cohen’s kappa as a measure of agreement. Stat. Med..

